# Tau phosphorylation induced by severe closed head traumatic brain injury is linked to the cellular prion protein

**DOI:** 10.1186/s40478-017-0435-7

**Published:** 2017-04-18

**Authors:** Richard Rubenstein, Binggong Chang, Natalia Grinkina, Eleanor Drummond, Peter Davies, Meir Ruditzky, Deep Sharma, Kevin Wang, Thomas Wisniewski

**Affiliations:** 1 Laboratory of Neurodegenerative Diseases and CNS Biomarker Discovery, Departments of Neurology and Physiology/ Pharmacology, SUNY Downstate Medical Center, 450 Clarkson Avenue, Box #1213, Brooklyn, 11203-2098 NY USA; 2 Center for Cognitive Neurology and Department of Neurology, New York University School of Medicine, Alexandria ERSP, 450 East 29th Street, New York, 10016 NY USA; 3 Litwin-Zucker Center for Research in Alzheimer’s Disease, Feinstein Institute for Medical Research, Manhasset, 11030 NY USA; 4 Program for Neurotrauma, Neuroproteomics and Biomarker Research, Departments of Psychiatry and Neuroscience, University of Florida, Gainesville, 32611 FL USA; 5 Center for Cognitive Neurology and Departments of Neurology, Pathology and Psychiatry, New York University School of Medicine, Alexandria ERSP, 450 East 29th Street, New York, 10016 NY USA

**Keywords:** Traumatic brain injury, Total Tau, Tau phosphorylation, GFAP, Cellular prion protein, Cognition, Immunohistochemistry

## Abstract

Studies in vivo and in vitro have suggested that the mechanism underlying Alzheimer’s disease (AD) neuropathogenesis is initiated by an interaction between the cellular prion protein (PrP^C^) and amyloid-β oligomers (Aβo). This PrP^C^-Aβo complex activates Fyn kinase which, in turn, hyperphosphorylates tau (P-Tau) resulting in synaptic dysfunction, neuronal loss and cognitive deficits. AD transgenic mice lacking PrP^C^ accumulate Aβ, but show normal survival and no loss of spatial learning and memory suggesting that PrP^C^ functions downstream of Aβo production but upstream of intracellular toxicity within neurons. Since AD and traumatic brain injury (TBI)-linked chronic traumatic encephalopathy are tauopathies, we examined whether similar mechanistic pathways are responsible for both AD and TBI pathophysiologies. Using transgenic mice expressing different levels of PrP^C^, our studies investigated the influence and necessity of PrP^C^ on biomarker (total-tau [T-Tau], P-Tau, GFAP) levels in brain and blood as measured biochemically following severe TBI in the form of severe closed head injury (sCHI). We found that following sCHI, increasing levels of T-Tau and P-Tau in the brain were associated with the PrP^C^ expression levels. A similar relationship between PrP^C^ expression and P-Tau levels following sCHI were found in blood in the absence of significant T-Tau changes. This effect was not seen with GFAP which increased within 24 h following sCHI and progressively decreased by the 7 day time point regardless of the PrP^C^ expression levels. Changes in the levels of all biomarkers were independent of gender. We further enhanced and expanded the quantitation of brain biomarkers with correlative studies using immunohisochemistry. We also demonstrate that a TBI-induced calpain hyperactivation is not required for the generation of P-Tau. A relationship was demonstrated between the presence/absence of PrP^C^, the levels of P-Tau and cognitive dysfunction. Our studies suggest that PrP^C^ is important in mediating TBI related pathology.

## Introduction

The cellular prion protein (PrP^C^) is a host-coded membrane-bound glycoprotein containing a glycosylphosphatidylinositol anchor. It is highly expressed in the central nervous system (CNS), especially in neurons [2, 21, 34, 41]. Although PrP^C^ is ubiquitous, involved in various biological processes and highly conserved between species, its major physiological role is still unknown. PrP^C^ is not essential for survival of mice as demonstrated by the PrP gene (Prnp) knockout mice (PrPKO). However, using PrPKO mice, numerous reports have described phenotypic changes including, but not limited to, abnormal circadian rhythm [51] and altered electrophysiology (increased seizure susceptibility, impaired long-term potentiation (LTP) [4, 5, 10, 12, 57]. PrP^C^ has also recently been linked to Alzheimer’s Disease (AD) neuropathology [20, 22, 23, 40].

Several studies have described a signaling cascade explaining the association of Aβ42 oligomers (Aβo) and PrP^C^ to AD pathology. Previous studies [23] have shown that PrP^C^ functions as a high-affinity receptor for Aβo and is also required for Aβo inhibition of hippocampal LTP. This suggests that PrP^C^ is required for the pathological effects of Aβo, such as inhibition of synaptic plasticity. Further studies have shown that Fyn kinase plays an important role in linking the Aβo-PrP^C^ interaction to disturbances in neuronal function. The connection between Aβo-PrP^C^ complexes at the cell surface and the intracellular Fyn kinase has been shown to require the metabotropic glutamate receptor, mGluR5 [53]. Taken together, the extracellular Aβo trigger neuronal signal transduction from PrP^C^ to mGluR5 to Fyn kinase. A loss of Fyn kinase function alleviates AD-related phenotypes in transgenic mice [8, 20, 36, 40] and the Aβo-PrP^C^-Fyn pathway has been shown to be relevant to AD in humans [40, 54]. Fyn regulates glutamate receptor trafficking and synaptic plasticity by phosphorylating N-methyl-D-aspartate glutamate receptor (NMDA-R) subunits NR2A and NR2B [14, 19, 30, 35, 49] which play an important role in learning and memory [26, 28]. Both PrP^C^ and Fyn have been localized to post-synaptic density (PSD)-containing brain fractions which is the primary post-synaptic site for signal transduction and processing in neurons.

In addition to its influence on NMDA-R, the Aβo-PrP^C^-Fyn complex results in phosphorylation of tau (P-Tau) [3, 22, 24]. P-Tau aggregates comprise neurofibrillary tangles (NFTs), one of the key hallmarks of AD.

Calpains are calcium-activated neutral proteases which, when transiently activated, are involved in normal cellular physiological processes. However, continued calpain hyperactivation following TBI is associated with neuropathology. Calpain-mediated proteolysis regulates the activity of key tau kinases, such as GSK-3 and cdk5, both of which promote tau phosphorylation and tau-associated neurodegeneration in vivo [11, 13, 31, 32]. Detection of stable proteolytic fragments of the cytoskeletal protein α-II-spectrin is specific for cleavage by calpains and is used as a marker of calpain activation in models of TBI. Post-traumatic inhibition of calpains is associated with attenuation of functional and behavioral deficits, axonal pathology, and cell death in animal models of TBI. We have previously demonstrated that WT, Tga20 and PrPKO mice subjected to sCHI resulted in calpain activation as demonstrated by the appearance of α-II-spectrin breakdown products (SBDPs). We further demonstrated that the administration of the calpain inhibitor, SNJ-1945, following sCHI reduced the levels of the SBDPs in all three mouse lines [39].

Trauma to the CNS is one of the most consistent risk factors for initiating the molecular cascades that result in the development of neurodegenerative diseases such as AD and, most recently, chronic traumatic encephalopathy (CTE). CTE is a progressive neurodegenerative disease that occurs in association with repetitive mild traumatic brain injury (rmTBI) in sports and military service [46]. The exact relationship between rmTBI, with or without symptomatic concussion, and CTE is unclear. It is possible that repetitive axonal injury initiates a series of metabolic, ionic, and cytoskeletal disturbances that trigger a pathological cascade leading to CTE in susceptible individuals. In most instances, the clinical symptoms of the disease begin after a long period of latency ranging from several years to several decades. The initial symptoms are typically insidious, consisting of irritability, impulsivity, aggression, depression, short-term memory loss and heightened suicide. The symptoms progress slowly over decades to include cognitive deficits and dementia. The pathology of CTE is characterized by the accumulation of P-Tau in neurons and astrocytes in a pattern that is distinct from other tauopathies, including AD [29]. The P-Tau abnormalities begin focally, as perivascular NFTs and neurites at the depths of the cerebral sulci, and then spread to involve superficial layers of adjacent cortex before becoming a widespread degeneration affecting medial temporal lobe structures, the diencephalon and brainstem. The majority of CTE cases (>85%) show abnormal accumulations of phosphorylated 43 kDa TAR DNA binding protein (TDP-43) that are partially colocalized with phosphorylated tau protein. Although definitive diagnosis of CTE requires neuropathological examination, a major current research goal is the identification of biomarkers for disease diagnosis and prognosis.

Although clinically and neuropathologically distinct, AD and CTE share some similarities. TBI causes Aβ aggregation in the brain along with low- and high-molecular-weight Aβo [59]. The formation and aggregation of Aβ into toxic species acutely after injury may play a role in secondary injury cascades after trauma. The tau isoform profile and phosphorylation state in CTE are similar to those in AD [43]. As described above, in vitro and in vivo studies have demonstrated that PrP^C^ can mediate the Aβo-Fyn kinase pathway and P-Tau accumulation in AD. We propose that the mechanisms associated with tau hyperphosphorylation in AD have commonality to TBI-induced tau pathology. If this hypothesis is true, it follows that PrP^C^ should play a role in mediating TBI related pathology. We therefore studied the effects of severe closed head traumatic brain injury (sCHI) on the generation of P-Tau and its relation to cognitive deficits using transgenic mouse lines having different levels of PrP^C^ expression.

## Materials and methods

### Mice

All animal studies were carried out in accordance with the National Institutes of Health guide for the care and use of laboratory animals and under the supervision and approval of the Institution Animal Care and Use Committee. Breeding pairs of PrP knockout (PrPKO) mice and PrP overexpressing (Tga20) mice were originally supplied by Dr. Charles Weissman (Scripps Research Institute, FL) and have been continually bred and maintained at SUNY Downstate Medical Center. The wild-type (WT) mice were generated and maintained in-house. The background genotype of all mice used in this study was C57BL/6 J x 129/SV. We have previously examined the levels of PrP^C^ expression and reported that, as expected, the PrPKO mice do not have detectable PrP^C^ while Tga20 mice express approximately eight times more PrP^C^ than WT mice [38].

### Animal model of TBI (mouse sCHI)

A baseline weight was obtained for WT, PrPKO and Tga20 mice prior to sCHI (or sham). Deep anesthesia was induced with isoflurane [3% in oxygen (1.0 l/min)] and maintained via a nose cone and isoflurane [2% in oxygen (1.0 l/min)]. The head of the mouse was fixed in a stereotaxic frame. Temperature was maintained at 36.5–37.5 °C using a temperature-controlled heating pad. Isoflurane anesthesia was maintained throughout the procedure. A cortical contusion was produced using an electromagnetic contusion device (Myneurolab, St. Louis, MO). A 5.0 mm diameter impactor tip was placed at a 10° angle 5.0 mm off the midline and 5.0 mm from the eyes of the mouse. sCHI was produced by a single impact to the skull at 6.3 m/s velocity and 3 mm depth and a dwell of 0.2 ms. After impact the mouse was removed from the device and allowed to recover in its home cage. A mouse was determined to have recovered from anesthesia if it regained its ability to right itself and ambulate. Control (sham) mice received the same procedure without the impact. Mice were weighed every day after sCHI (or sham). For each round of experiments, groups of mice (8 mice/group at approximately 3.5 months of age) were subjected to sCHI (or sham) and used first for cognitive assessment at 7 days post sCHI (or sham). Following an additional week these same groups of mice were anesthetized using isoflurane (4% for induction and 3% for maintenance) in oxygen (0.8 L/min) and transcardially perfused with 10% sucrose solution in PBS, followed by 4% paraformaldehyde solution in PBS. Following fixation, brains were prepared for histological analysis and immunohistochemistry (IHC).

Additional groups of mice (at approximately 3.5 months of age) expressing varied levels of PrP^C^ (WT, PrPKO and Tga20 mice) were subjected to sCHI (or sham treated). At 1, 3 and 7 days post-trauma these mice were euthanized with isoflurane and blood was collected by cardiac exsanguination. Blood was collected in heparanized tubes, centrifuged and plasma stored frozen. Mouse brains were removed, dissected and frozen. Plasma and cortical brain tissue were analyzed for TBI biomarkers [Tau, glial fibrillary acidic protein (GFAP)]. The numbers of mice used for each mouse strain and condition (sCHI vs sham) are indicated in the figure legends.

### Calpain inhibitor SNJ-1945 preparation and administration in vivo

Calpain was inhibited by intraperitoneal (i.p.) administration of SNJ-1945 (100 mg/kg) at 3 and 24 h post sCHI (or sham). SNJ-1945 was prepared as a stock concentration of 3 mg/ml in 0.5% carboxymethyl cellulose (CMC) in distilled water. To prepare the SNJ-1945 stock suspension, the weighed amount of SNJ-1945 is ground up with a mortar and pestle in the presence of 200 μl 0.5% CMC. When the prepared SNJ can pass through a 22 g needle add 0.5% CMC to the desired final volume. SNJ-1945 was prepared fresh as needed. The numbers of mice of each strain that were SNJ-1945-treated or untreated are indicated in the figure legends.

### Post-sCHI behavior assessment

Behavior and cognitive assessment were analyzed using an Interactive Tracking System (BioSignal Group Corp., Brooklyn, NY) capable of monitoring and measuring cognitive deficits using active place avoidance (APA) and conflict avoidance [15, 39].

In brief, a rotating behavioral arena is placed in a rectangular room with visual cues on the walls. A computer-controlled Firewire camera mounted above the arena monitored the position of a mouse. A computer-defined segment of the arena consisted of a do-not-enter shock zone with a 0.2 mA shock applied at 500 ms interval after entry into this zone. The number of times a mouse entered and remained in the shock zone was computed by Track Analysis software (BioSignal Group Corp., Brooklyn, NY).

Beginning 7 days after sCHI (or sham), mice had pre-training (open field) sessions in the rotating arena with the shock zone turned off. The mice were then subjected to APA in 4 sessions, 10 min each with a 50 min inter-trial interval with the shock zone turned on. A 500 ms shock was applied after a mouse entered the shock zone. Additional shocks were administered every 1.5 s until the mouse vacated the shock zone. The number of entrances into the shock zone will measure avoidance and the distance traveled will measure locomotion. Compared to sham mice, the sum of shock zone entrances of sCHI mice after 4 trials is inversely correlated to cognitive function. On the following day, the mice were subjected to APA conflict learning in which the shock zone was shifted 180° from its original location. Conflict learning tested whether avoidance memory between the two shock zone locations conflicted.

### T-Tau, P-Tau and GFAP quantitation by laser-based immunoassay

The anti-Tau monoclonal antibodies (Mabs) used were previously described [1]. Biomarkers in blood were detected using enhanced immunoassay using multi-arrayed fiber optics coupled to rolling circle amplification (RCA) (a-EIMAF) while a similar procedure in the absence of RCA (EIMAF) was used for brain tissue. For a-EIMAF, high-binding 96-well microtiter plates were coated with Mab DA31 at 6 μg/ml final concentration for T-Tau, Mab CP13 (pSer-202) for P-Tau and a combination of Mabs 2E1, 1B4, 4A11 for GFAP (BD Pharmingen (Franklin Lakes, NJ). Following an overnight incubation at 40 °C, unoccupied binding sites were blocked for 1 h with casein. A 100 μl aliquot of diluted (10^−7^) cortex or blood (plasma at 1:40 dilution is used to avoid matrix effects) sample was added, incubated and followed by the addition of a biotinylated detection Mab DA9 (100 μl at 4 μg/ml final Mab concentration) for Tau and a rabbit anti-GFAP polyclonal Ab (Abcam, Cambridge, MA) for GFAP. Five 10 min washes with PBST were followed with the addition of 100 μl of streptavidin (5 μg/ml) per well and incubation for 1 h at 37 °C. A biotinylated DNA primer (5’-TTTTTTTGTCCGTGCTAGAAGGAAACAGTTAC-3’) (100 μl at 4 μg/ml) was added and the plate incubated for 1 h at 37 °C. Following the addition of a T4-DNA ligase-pretreated IgE DNA template (1 mg/ml), amplification was initiated by adding 100 μl of reaction mixture consisting of: φ29 DNA polymerase reaction buffer, bovine serum albumin, nucleotide triphosphates supplemented with dUTP-Texas Red, and φ29 DNA polymerase. Incubation for several hrs was followed by PBST washes, addition of 1 N NaOH, neutralization with 1 M Tris-HCl, pH 7.5, heat treatment (100 °C for 15 min) and fluorescence analysis. For direct, non-amplified detection and relative quantitation of Tau and GFAP in brain, EIMAF was performed as detailed previously and briefly described here [7]. Diluted brain tissue lysates were added to the capture Ab followed by the biotinylated detection Mab. Following a 1 h incubation, streptavidin conjugated to Rhodamine Red X (1:1000) (Invitrogen) was added and incubated for 1 h. The wells were washed with TBS containing Tween-20, then treated with NaOH and neutralized. A 90 μl sample was drawn up into a 100 μl Microcap (Drummond Scientific) micro-capillary tube, which was then inserted into a specially designed tube sample holder for laser excitation and emission quantitation. Each EIMAF and a-EIMAF sample was tested in triplicate and, depending on available sample volumes, duplicated in independent experiments.

Standard curves were generated and used to convert a-EIMAF and EIMAF voltage readings to actual T-Tau, P-Tau and GFAP concentrations. Recombinant human Tau protein (N2R4 isoform with 441 residue) (R-peptide Co.) was used as T-Tau standard. Tau tubulin kinase 1 (TTBK1)-phosphorylated recombinant Tau protein (Tau protein co-expressed with TTBK1 in E. coli cells) (SignalChem Co.) was used as P-Tau (pSer202). T-Tau and P-Tau preparations were > 95% pure based on major T-Tau or P-Tau band intensity over background and other minor bands as determined by SDS-PAGE followed by Coomassie Blue total protein staining and densitometric scanning quantitation with NIH Image J software.

### Neuropathological analysis of brain by IHC

Throughout this project, behavioral studies were performed on mice at 7 days post neurotrauma and at 14 days post trauma, the mouse brains were analyzed by IHC. Mice were subjected to deep anesthesia, perfused with 4% paraformaldehyde (PFA), their brains removed and stored in 4% PFA. The fixed brains were paraffin-embedded and sectioned. Nine micron sagittal sections were collected onto microscope slides (either 4 or 6 sections/slide) half of the sections containing the injured side of the brain and the other half containing the uninjured side of the brain. Assessment of mouse brain sections using fluorescent IHC and hematoxylin and eosin staining was performed by a blinded investigator to determine the presence and location of the injury site. It was found that regardless of mouse strain (WT, Tga20, PrPKO), those mice subjected to neurotrauma showed gliosis consistent with head injury to an extent that enabled the blinded investigator to confidently determine the injured hemisphere. In a number of cases there was evidence of extensive contralateral gliosis in addition to gliosis surrounding the injured area. The injured region was consistently present in the visual cortex and gliosis was often present in the underlying hippocampal formation and neighboring somatosensory and motor cortices.

Fluorescent IHC (F-IHC) was performed to quantify protein staining using standard protocols. Sections were stained with primary antibodies against GFAP (Dako, Santa Clara, CA), IBA1 (Wako, Richmond, VA), PrP^C^ (Mab 6D11; Santa Cruz, Dallas, TX), P-Tau (CP13; provided by Peter Davies), T-Tau (Tg5; provided by Peter Davies), myelin basic protein (MBP) (BioLegend, San Diego, CA) and microtubule associated protein 2 (MAP2) (BD Biosciences, San Jose, CA). Briefly, sections were dewaxed and rehydrated using xylene and decreasing concentrations of ethanol. Antigen retrieval was performed by boiling sections in citrate buffer for 20 min (10 mM sodium citrate, 0.05% Tween-20, pH 6). Slides were then blocked with either 10% normal goat serum (GFAP, IBA1, 6D11, CP13, MBP and MAP2) or mouse-on-mouse (M.O.M.) blocking solution (Vector Labs, Burlingam, CA) (Tg5) for 1 h at room temperature. Sections were then incubated with primary antibody solutions diluted in either 4% normal goat serum (GFAP, 1:1000; IBA1, 1:500; 6D11, 1:2000; CP13, 1:500; MBP, 1:1000; MAP2, 1:2000) or M.O.M. protein concentrate (Tg5, 1:100) overnight at 4 °C. This was followed by incubation with appropriate fluorescent conjugated secondary antibodies (Jackson ImmunoResearch, West Grove, PA) diluted 1:500 in PBS for 2 h at room temperature, and then incubation with Hoechst 33342 (Sigma Chemical Co., St. Louis, MO) for 10 min at room temperature to label nuclei. Slides were cover-slipped and fluorescent imaging of the whole slide was performed at 20x magnification using a NanoZoomer HT2 (Hamamatsu) whole slide scanner using the same settings for all slides.

Initial F-IHC semi-quantification was performed to determine the presence of injury (yes/no result). Quantification was performed on 6 sections/animal; for injured animals this included 3 sections of the injured (ipsilateral) hemisphere and 3 sections of the contralateral hemisphere. For quantification, 4x magnification images containing the cortex and the underlying hippocampus were collected from each section using the NanoZoomer full slide scanner viewing software. Quantification of fluorescent staining was performed using ImageJ software. For quantification of 6D11 and Tg5 staining, the average staining intensity in the cortex was determined. For GFAP, IBA1, MBP and MAP2 the percentage of staining burden was quantified in the cortex. This was performed by defining the cortex as the region of interest (taking care not to include the edge of the tissue or any folds or damage in the cortex) and applying a threshold to the GFAP, IBA1, MBP and MAP2 staining to determine the number of immuno-positive pixels present in the whole cortex. The positive staining threshold was determined for each immunostain as the average optimal threshold that allowed discrimination between staining from background levels for all images included in the analysis. The same threshold was then applied to all brains immunostained with the same antibody. CP13 staining in the cortex was semi-quantified on a scale of 0–2 (0 = no staining, 1 = obvious CP13 staining, 2 = bright CP13 staining in cortex).

### Statistical analysis

Statistics was performed using GraphPad Prism (Version 7.02). For analysis of biochemical studies, one-way ANOVA with Tukey post-hoc for multiple comparisons was used to determine whether significant differences existed in biomarker concentrations between different mouse strains when subjected to sCHI. However, when determining whether significant differences existed in biomarker concentrations between the different mouse strains and at different time points in which sCHI and sham treatments were also compared, two-way ANOVA with Tukey post-hoc for multiple comparisons was used. For behavioral studies, one-way ANOVA with Student Newman Keul’s post-hoc for multiple comparison was used to identify significant differences between groups. In the case of the neuropathological studies, one-way ANOVA with Tukey post-hoc analysis was used to identify significant differences between experimental groups. Significant differences between experimental groups that are reported are limited to those between sham and sCHI for a particular genotype and between WT, Tga20 and PrPKO sCHI groups. In all cases *p* < 0.05 was considered to be significant.

## Results

WT, Tga20 and PrPKO mice developed two injury syndromes as a result of sCHI. Following the injury, mice could be divided into two groups based on the time period for the righting reflex and the extent of apnea (i.e. loss of consciousness). Mice were designated sCHI-1 if breathing was initiated spontaneously within 30 s and the righting reflex was within 10 min whereas mice categorized as sCHI-2 required the aid of administered oxygen and had a righting reflex greater than 10 min. Greater than 85% of the all mice in each of the three groups (WT, Tga20, PrPKO) were sCHI-2. As a result, the studies reported here were focused solely on sCHI-2 mice (which are referred to in this manuscript as simply, sCHI). As we previously reported [39], sCHI did not significantly alter the PrP^C^ expression levels in cortex, hippocampus and cerebellum of either WT or Tga20 mice compared to the sham-treated controls.

### Biochemical analysis of Tau and GFAP

**Fig. 1:**
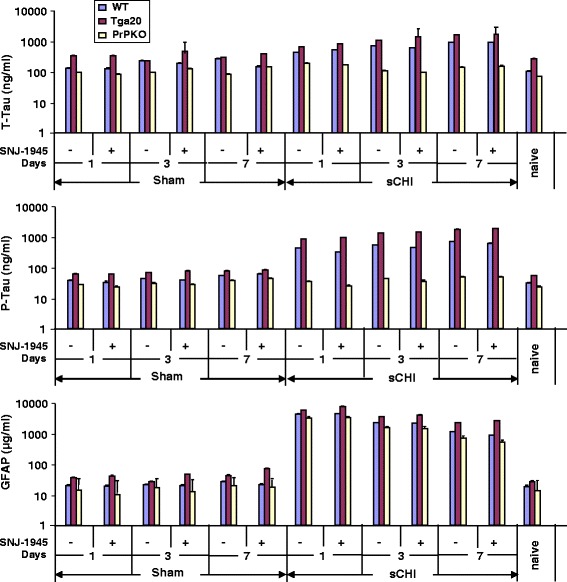
Detection of *T-Tau*, *P-Tau* and *GFAP* in the cortical brain regions of WT, Tga20 and PrPKO mice following sCHI (or sham). EIMAF was used to measure T-Tau, P-Tau and GFAP in a 10^−7^ dilution of WT, Tga20 and PrPKO mouse brain cortices at 1, 3 and 7 days post sCHI (or *sham*) in untreated and SNJ-1945 (calpain inhibitor)-treated animals. At each time point, and for each of the three mouse strains, 10 sham mice (5 without and 5 with SNJ-1945) and 10 sCHI mice (5 without and 5 with SNJ-1945) were used. Values are expressed as mean ± SD

The concentrations of T-Tau, P-Tau and GFAP were determined in the brains of the three mouse strains following sCHI and sham. Compared to sham-treated mice, following sCHI of WT mice the levels of T-Tau and P-Tau significantly increased in the cortex at the three time points (T-Tau: Tukey *p* < 0.0001 at days 1, 3 and 7; P-Tau: Tukey *p* < 0.0001 for days 1, 3 and 7) examined post impact (Fig. 1). The T-Tau concentrations progressively increased from 388 ± 5 ng/ml at day 1 to 811 ± 4 ng/ml at day 7 post sCHI. The patterns of increasing T-Tau in Tga20 mice were generally similar to that described for WT mice but the actual biomarker concentrations for sham and sCHI Tga20 mice were higher than for WT mice. Following sCHI, the T-Tau concentrations increased from 581 ± 13 ng/ml on day 1 to 1412 ± 10 ng/ml by day 7 post sCHI which were significantly greater than both the sham-treated Tga20 mice at all time points (Tukey *p* < 0.0001) and the sCHI (and sham) WT mice (Tukey *p* < 0.0001). Interestingly, PrPKO mice subjected to sCHI displayed a different biomarker pattern than both WT and Tga20 mice (Fig. 1). Following sCHI the concentrations of T-Tau increased slightly at day 1 to 176 ± 7 ng/ml compared to the sham mice (86 ± 2 ng/ml) and then by day 7 decreased 128 ± 4 ng/ml compared to 77 ± 4 ng/ml in sham mice. At all time points post sCHI, the concentrations of T-Tau in PrPKO mice was significantly less than WT mice (Tukey *p* < 0.0001) and Tga20 mice (Tukey *p* < 0.0001).

The levels of P-Tau in WT mouse brain significantly (Tukey *p* < 0.0001) exceeded those of T-Tau post sCHI at all three time points from 536 ± 5 ng/ml at day 1 and continued increasing to 898 ± 9 ng/ml at day 7. Unrelated studies quantitating Tau in CSF also reported that P-Tau levels were higher than the levels of T-Tau [56]. Similar to WT mice, the P-Tau concentrations in sCHI-treated Tga20 cortex continued to increase significantly (Tukey *p* < 0.0001) from day 1 (1090 ± 13 ng/ml) and continued to rise at 3 days (1684 ± 32 ng/ml) and 7 days (2293 ± 23 ng/ml) post sCHI. In contrast, the P-Tau concentrations in the cortex of PrPKO mouse brain did not demonstrate any significant increase in either sham (31–43 ng/ml) or sCHI (39–56 ng/ml) subjected mice compared to the WT and Tga20 post sCHI (Tukey *p* < 0.0001).

The GFAP concentrations in the cortices of the three mouse strains were also examined after sCHI (Fig. 1). GFAP had a maximum increase at 1 day post injury (3220 ± 29 μg/ml) and continued to significantly decline thereafter at 3 days (1710 ± 18 μg/ml) and 7 days (892 ± 11 μg/ml) (Tukey *p* < 0.0001). A similar pattern of maximum GFAP concentrations at day 1 post sCHI which continued to decrease by day 7 was observed in both Tga20 (4190 ± 59 μg/ml, 2640 ± 45 μg/ml and 1710 ± 30 μg/ml at days 1, 3 and 7, respectively) and PrPKO mice (2400 ± 24 μg/ml, 1180 ± 14 μg/ml) and 561 ± 8 μg/ml at days 1, 3 and 7, respectively). The GFAP concentrations in the cortices of the three mouse strains significantly (Tukey *p* < 0.0001) differed from each other at each time point post sCHI.

We also examined the influence of calpain activity on the levels of T-Tau, P-Tau and GFAP after sCHI by treating the impacted mice with the calpain inhibitor, SNJ-1945. Compared to SNJ-untreated mice, treating the three mouse strains with SNJ-1945 had no significant effect on the patterns of T-Tau, P-Tau and GFAP throughout the entire 7 day study (Fig. 1).

**Fig. 2:**
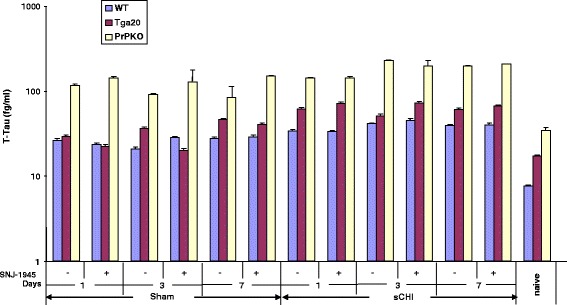
Detection of *T-Tau* in plasma from *WT*, *Tga20* and *PrPKO* mice following sCHI (or *sham*). a-EIMAF was used to measure T-Tau in plasma at 1, 3 and 7 days post sCHI (or *sham*) from WT, Tga20 and PrPKO mice that were untreated or treated with the calpain inhibitor SNJ-1945. The numbers of mice used at each time point were: WT - 8 sham (4 without and 4 with *SNJ-1945*) and 9 sCHI (5 without and 4 with *SNJ-1945*), Tga20 - 8 sham (4 without and 4 with *SNJ-1945*) and 9 sCHI (5 without and 4 with *SNJ-1945*), PrPKO - 8 sham (4 without and 4 with SNJ-1945) and 9 sCHI (5 without and 4 with *SNJ-1945*)

Blood was analyzed for the presence of biomarkers in a manner similar to brain. Generally, changes in biomarker levels as a result of sCHI were less pronounced in blood than in brain. Furthermore, we extended these studies to assess whether there were gender differences in blood-based biomarker responses to sCHI. Since we did not find any gender-specific differences in the concentrations of T-Tau, P-Tau and GFAP at each time point in sham and sCHI-treated mice, regardless of mouse strain or calpain activity (data not shown), we combined the data for both genders of each strain. Following sCHI of WT mice, the T-Tau concentrations in blood did not change significantly at all time points (34–40 fg/ml) (Fig. 2). The general pattern of the changes in plasma T-Tau concentrations from PrP^C^-overexpressing Tga20 mice post sCHI were found to be similar to WT mice. However, the absolute biomarker levels in Tga20 were significantly (Tukey *p* < 0.0001) greater than in WT mice (Fig. 2). Between days 1 and 7, the T-Tau concentrations in the sham-treated mice ranged from 28 ± 1.2–58 ± 2.3 fg/ml. Following sCHI, T-Tau concentrations increased between days 1 and 7 to 56 ± 2.6–78 ± 2.6 fg/ml. Furthermore, in contrast to brain, the T-Tau concentrations in PrPKO sham and sCHI mice were significantly (Tukey *p* < 0.0001) higher than in the corresponding WT and Tga20 mice and remained elevated throughout the 7 day time course (sham: 84 ± 1.7–134 ± 3.1 fg/ml and sCHI: 124 ± 3.9–240 ± 8.5 fg/ml. SNJ-1945 did not significantly alter the T-Tau concentrations in all mouse strains.

**Fig. 3:**
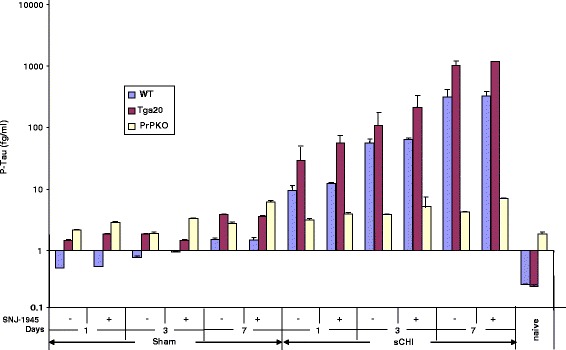
Detection of *P-Tau* in plasma from *WT*, *Tga20* and *PrPKO* mice following sCHI (or *sham*). a-EIMAF was used to measure P-Tau in plasma at 1, 3 and 7 days post sCHI (or *sham*) from Tga20 mice that were untreated or treated with the calpain inhibitor SNJ-1945. The numbers of mice used at each time point were: WT - 8 sham (4 without and 4 with *SNJ-1945*) and 9 sCHI (5 without and 4 with *SNJ-1945*), Tga20 - 8 sham (4 without and 4 with *SNJ-1945*) and 9 sCHI (5 without and 4 with *SNJ-1945*), PrPKO - 8 sham (4 without and 4 with *SNJ-1945*) and 9 sCHI (5 without and 4 with *SNJ-1945*)

However, the P-Tau levels in plasma from WT mice increased significantly at each time point (Tukey *p* < 0.0001) following sCHI (compared to sham) beginning at day 1 (0.5 ± 0.02 fg/ml in sham vs. 9.5 ± 0.22 fg/ml in sCHI) and continuing to day 7 (1.5 ± 0.1 in sham vs. 307 ± 5.5 fg/ml in sCHI) (Fig. 3). In the Tga20 mice, the sCHI-induced rise in plasma P-Tau concentrations were more dramatic than the WT mice (Tukey *p* < 0.0001) and progressively increased from 29 ± 0.7 (day 1) - 1018 ± 15 (day 7). However, in contrast to WT and Tga20 mice, plasma P-Tau levels of PrPKO mice following sCHI did not display a robust increase over the course of the study (3.2 ± 0.2 (day 1)–6.2 ± 0.2 (day 7) and was significantly less than the WT and Tga20 mice (Tukey *p* < 0.0001). This is similar to the overall P-Tau patterns observed in brain following sCHI.

**Fig. 4:**
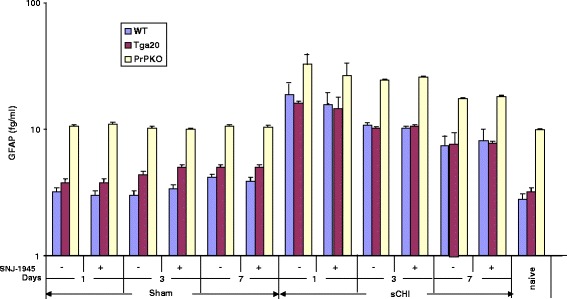
Detection of *GFAP* in plasma from *WT*, *Tga20* and *PrPKO* mice following sCHI (or *sham*). a-EIMAF was used to measure GFAP in plasma at 1, 3 and 7 days post sCHI (or *sham*) from PrPKO male and female mice that were untreated or treated with the calpain inhibitor SNJ-1945. The numbers of mice used at each time point were: WT - 8 sham (4 without and 4 with *SNJ-1945*) and 9 sCHI (5 without and 4 with *SNJ-1945*), Tga20 - 8 sham (4 without and 4 with *SNJ-1945*) and 9 sCHI (5 without and 4 with *SNJ-1945*), PrPKO - 8 sham (4 without and 4 with *SNJ-1945*) and 9 sCHI (5 without and 4 with *SNJ-1945*)

A short-lived GFAP increase in WT mouse plasma was measured within 24 h post sCHI (20 ± 0.6 fg/ml vs. 3.2 ± 0.3 fg/ml in sham mice) but then declined throughout the remainder of the study to 8.8 ± 0.3 fg/ml (compared to 4.2 ± 0.3 fg/ml in sham WT mice) by day 7 (Fig. 4). This GFAP pattern in plasma from WT mice was similar to that in the WT mouse brain. In Tga20 mice, there was an increase in GFAP measured in plasma at day 1 post sCHI (16 ± 0.5 fg/ml) which decreased by day 7 (8.8 ± 0.5 fg/ml). In PrPKO mice, the levels of GFAP increased at 1 day after sCHI and followed a similar decreasing pattern, albeit higher absolute values, compared to WT and Tga20 mice post sCHI. The GFAP concentrations in the PrPKO mice were significantly greater than both the WT and Tga20 mice at each time point post sCHI (Tukey *p* < 0.0001). Any sCHI-induced increases in calpain activity [39] did not significantly influence plasma GFAP levels in all mouse strains (Fig. 4).

### Behavioral analysis

**Fig. 5:**
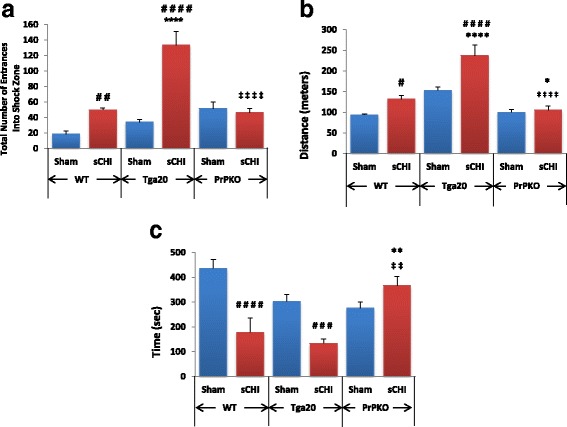
Behavioral Assessment of Mice Following sCHI (or *sham*). Groups of mice from each of the three mouse strains were subjected to sCHI (or sham) and evaluated for cognitive deficits, as described in Materials and Methods, beginning at 7 days post neurotrauma. learning and memory was assessed were performed on 8 mice per group. The total number of shock zone entrances in four trials (**a**) measured the ability of the mice to learn the shock zone location. Cognitive deficits are represented by an increase in the shock zone entrances. Significant differences were observed between: WT Sham vs WT sCHI (^##^ *p* = 0.0052), Tga20 Sham vs Tga20 sCHI (^####^ *p* = 0.0001), WT sCHI vs Tga20 sCHI (^****^ *p* = 0.0001) as well as Tga20 sCHI vs PrPKO sCHI (^‡‡‡‡^ *p* = 0.0001). The total distance traveled in four trials (**b**) assesses an aversively conditioned ability to inhibit movement. An increase in the total distance traveled suggests cognitive impairment. Significant differences were found between WT Sham vs WT sCHI (^#^ *p* = 0.0493), Tga20 Sham vs Tga20 sCHI (^####^ *p* = 0.0001), WT sCHI vs Tga20 sCHI (^****^ *p* = 0.0001), WT sCHI vs PrPKO sCHI (^*^ *p* = 0.0497) as well as Tga20 sCHI vs PrPKO sCHI (^‡‡‡‡^ *p* = 0.0001). The maximum time of avoidance on the 4^th^ trial (**c**) assayed the ability of the mouse to retain the shock zone location from the previous trial. An increase in the maximum time of avoidance suggests superior learning ability of mice due to training. Significant differences were found between WT Sham vs WT sCHI (^####^ *p* = 0.0001), Tga20 Sham vs Tga20 sCHI (^###^ *p* = 0.001), WT sCHI vs PrPKO sCHI (^**^ *p* = 0.0063) as well asTga20 sCHI vs PrPKO sCHI (^‡‡^ *p* = 0.0047)

Cognitive function was assessed in the sham and sCHI-treated mice to compare: 1) each mouse strain, 2) gender differences, 3) influence of calpain activation. Neither gender differences nor significant differences in SNJ-1945 effects were observed in WT, Tga20 and PrPKO strains (data not shown). Overall, Tga20 sCHI mice displayed greatest cognitive deficits as measured by the number of shock zone entrances, maximum distance traveled within the arena and the amount of time avoiding the shock zone (Fig. 5a–c). During APA, the rotating arena has a 60° stationary shock zone that remains fixed in its position within the moving arena. Assaying the total number of shock zone entrances is indicative of shock zone avoidance while the distance traveled measured inhibition of movement under adverse conditions. The number of shock zone entrances during APA testing relies on hippocampus-dependent short-term memory. Compared to WT and PrPKO sham and sCHI groups, Tga20 mice subjected to sCHI displayed a significant increase in the total number of entrances into the shock zone (Fig. 5a). WT sCHI mice had significantly more entrances into the shock zone as compared to WT sham. On the other hand, total number of entrances into the shock zone of PrPKO sCHI mice were not significantly different from the PrPKO sham mice. Not only did the Tga20 sCHI mice have the highest number of shock zone entrances, but also the highest values of distance traveled within the arena indicating uninhibited movement within the arena and failure to recall aversive areas (Fig. 5b) and the least amount of time to avoid the shock zone (i.e., did not remember to avoid the shock zone) on the fourth trial (Fig. 5c). On the other hand, not only did PrPKO sCHI mice have the least number of shock zone entrances but also the least amount of distance traveled (i.e., maintain a distance away from the shock zone rather than randomly exploring the arena) and the most time avoiding the shock zone. The combination of all these parameters supports the findings that PrPKO mice maintained the highest levels of learning and memory, followed by WT sCHI mice while Tga20 had the lowest. Furthermore, based on the specific and random positional movement of the mice in the arena and total radial distance traveled within the arena, males exhibited a lower tolerance to stress and higher anxiety than females irrespective of the levels of PrP^C^ expression (data not shown).

### Neuropathology

**Fig. 6:**
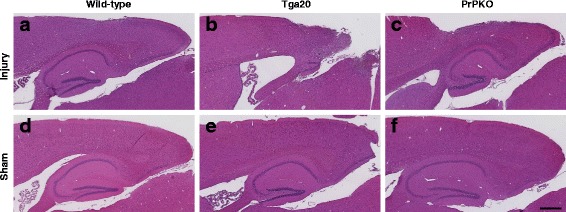
H + E staining showing the morphological changes in the brain after sCHI (**a**–**c**) in comparison to sham animals (**d**–**f**). *Scale bar* = 500 μm

**Fig. 7:**
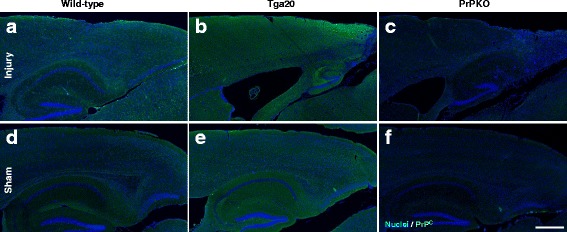
IHC using anti-PrP Mab 6D11 to assess the levels of PrP^C^ in brains from *WT, Tga20* and *PrPKO* mice at 14 days post sCHI. The amount of PrP^C^ was higher in Tga20 mice (**b**, **e**) in comparison to WT mice (**a**, **c**), and there was no PrP^C^ observed in PrPKO mice (**c**, **f**). *Scale bar* = 500 μm

**Fig. 8:**
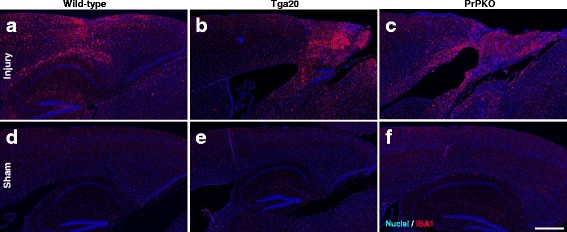
IHC using IBA1 to assess microgliosis in brains from *WT*, *Tga20* and *PrPKO* mice at 14 days post sCHI. *Scale bar* = 500 μm

**Fig. 9:**
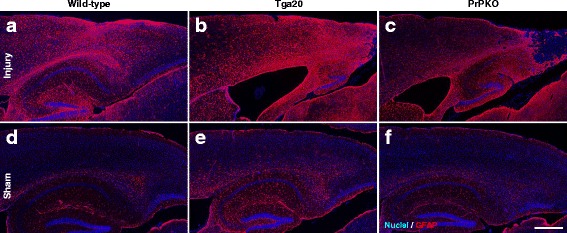
IHC using GFAP to assess astrocytosis in brains from *WT*, *Tga20* and *PrPKO* mice at 14 days post sCHI. *Scale bar* = 500 μm

**Fig. 10:**
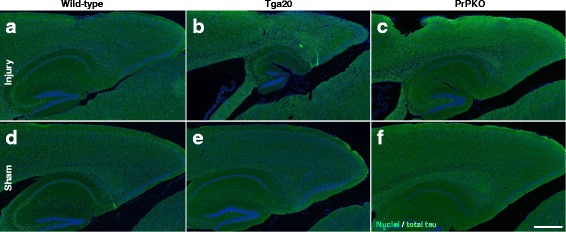
IHC of brains from *WT*, *Tga20* and *PrPKO* mice at 14 days post sCHI using a Mab to T-Tau. The intensity of T-Tau immunostaining was determined in the cortex. The T-Tau staining was similar for all mouse lines and was not altered following sCHI. *Scale bar* = 500 μm

**Fig. 11:**
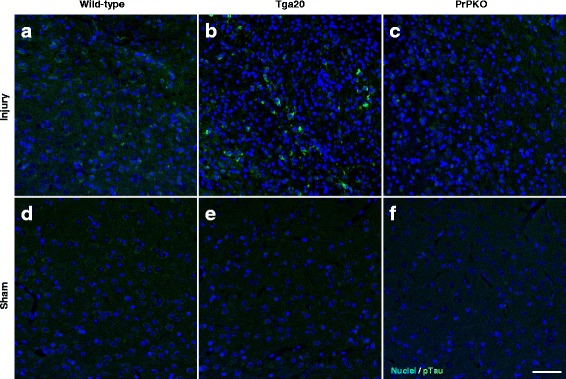
IHC of brains from *WT*, *Tga20* and *PrPKO* mice at 14 days post sCHI using the CP13 Mab to P-Tau. *Scale bar* = 50 μm

**Fig. 12:**
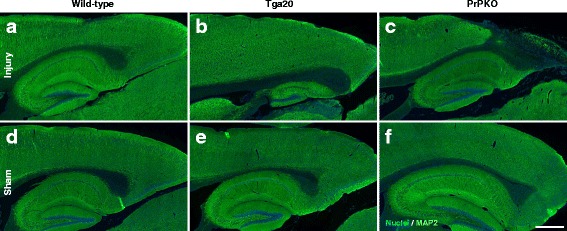
IHC of brains from *WT*, *Tga20* and *PrPKO* mice at 14 days post sCHI using a Mab to MAP2. The intensity of MAP2 immunostaining was determined in the cortex. The MAP2 staining was similar for all mouse lines and was not altered following sCHI. *Scale bar* = 500 μm

**Fig. 13:**
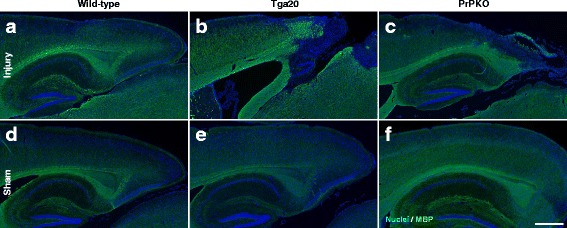
IHC of brains from *WT*, *Tga20* and *PrPKO* mice at 14 days post sCHI using a Mab to MBP. The intensity of MBP immunostaining was determined in the cortex. The MBP staining was similar for all mouse lines and was not altered following sCHI. *Scale bar* = 500 μm

**Fig. 14:**
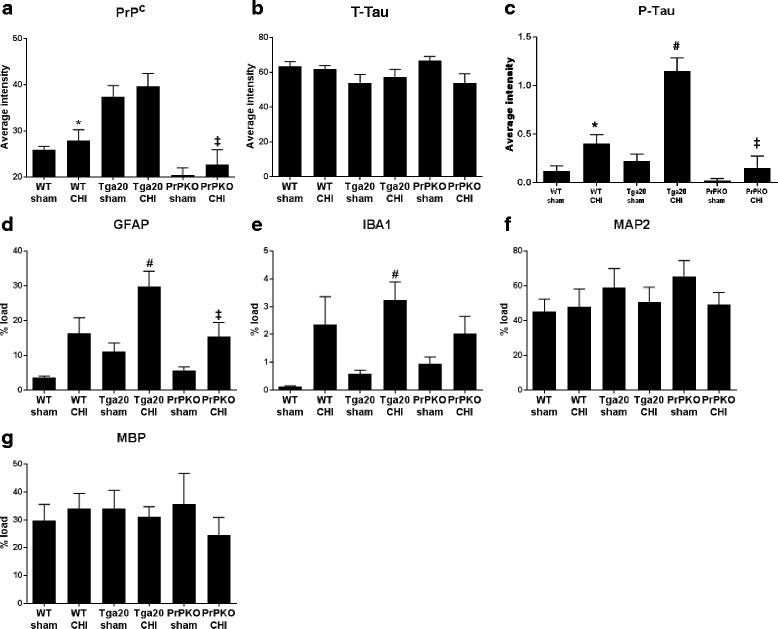
Quantification of IHC staining in the cortex for PrP^C^ (**a**), T-Tau (**b**), P-Tau (**c**), GFAP (**d**), IBA1 (**e**), MAP2 (**f**) and MBP (**g**). Quantification of PrP^C^ and T-Tau was determined as the average staining intensity in the cortex. Semi-quantification of P-Tau staining localized to the injury zone was analyzed using a semi-quantification rating of P-Tau intensity on a scale of 0–2 (2 being maximum staining). Quantification of GFAP, IBA1, MAP2 and MBP was determined as the percentage burden of immunopositive pixels in the cortex. Significant differences between groups were determined using one-way ANOVA with Tukey post-hoc test for multiple comparisons. For all graphs, # indicates significant differences (*p* < 0.05) between Tga20 sham and Tga20 sCHI; * Shows significant differences (*p* < 0.05) between WT sCHI and Tga20 sCHI; ‡ Shows significant differences (*p* < 0.05) between Tga20 sCHI and PrPKO sCHI

Assessment of the stained sections was performed by a blinded investigator to determine the presence and location of the injury site. It was found that the WT, Tga20 and PrPKO injured animals showed gliosis consistent with head injury to an extent that enabled the blinded investigator to confidently determine the injured hemisphere. In a number of cases there was evidence of extensive contralateral gliosis in addition to gliosis surrounding the injured region. The injury zone was also obvious on H + E stained sections (Fig. 6). The injured region was consistently present in the visual cortex and gliosis was often present in the underlying hippocampal formation and neighboring somatosensory and motor cortices. PrP^C^ was quantified as the total average staining intensity in the cortex (Fig. 7). As expected, there was significantly more PrP^C^ immuno-positive staining in Tga20 mice in comparison to WT mice (Tukey; *p* < 0.05), and PrPKO mice (Tukey; *p* < 0.0001). Interestingly, PrP^C^ levels in the cortex was unaffected by injury in all experimental groups (Fig. 14a). This finding is similar to what we have demonstrated previously using western blotting in that the expression levels of PrP^C^ are unchanged post sCHI [39]. As expected, sCHI resulted in significant gliosis in injured brains. This was most evident inside the injury zone, but there was also evidence of increased distal gliosis in the contralateral cortex after sCHI. The extent of microgliosis was determined by immunostaining with IBA1, which is a pan-microglial marker. Microgliosis was evident in the localized region affected by sCHI in all groups (Fig. 8). However, widespread microgliosis that was quantified in the cortices ipsilateral and contralateral to the injury was only significantly increased in Tga20 sCHI mice in comparison to Tga20 sham mice (Tukey; *p* < 0.05). In comparison there was no significant difference between injured and sham WT mice or PrPKO mice (Fig. 14e). The extent of increased astrogliosis in each experimental group followed a similar pattern to microgliosis. GFAP immunostaining was increased in the region localized to the brain injury in all groups (Fig. 9). The regional pattern of increased GFAP was somewhat different from the increased IBA1, in that brain injury produced a more widespread GFAP increase throughout the cortex and hippocampus in the injured hemisphere, while increased IBA1 immunoreactivity was more limited to the injury zone. This difference in the regional pattern of increased microgliosis and astrocytosis is evident comparing Figs. 8a–c and 9a–c. Widespread increases in GFAP was only significantly increased after injury in Tga20 mice, in comparison to control mice (Tukey; *p* < 0.01), and not after injury in WT or PrPKO mice (Fig. 14d). In addition, widespread GFAP immunostaining was significantly higher in Tga20 injured mice in comparison to the PrPKO injured mice (Tukey *p* < 0.05), showing that there was a greater astrocyte response to sCHI in mice overexpressing PrP^C^. T-Tau expression was ubiquitous in neurons throughout the brain, primarily present in neuronal cytoplasm and the primary portion of some axons. The amount of T-Tau was similar in WT, Tga20 and PrPKO mice (Fig. 10). Injury did not significantly alter T-Tau expression in the cortex in any experimental group (Fig. 14b). In contrast, the production of P-Tau after injury significantly differed between groups (one-way ANOVA *p* < 0.0001). P-Tau was observed as bright punctate staining around and within the injury site; predominantly in the cortex and the fornix. Fainter P-Tau staining was also observed in neurons throughout the cortex (see Fig. 11 showing an area of cortex just proximal to the injury zone). Injury resulted in significantly increased P-Tau expression in Tga20 mice in comparison to sham Tga20 mice (Tukey; *p* < 0.0001), while there was no significant increase in P-Tau expression after injury in WT or PrPKO mice (Fig. 14c). P-Tau expression was also significantly higher after injury in Tga20 mice in comparison to both WT mice (Tukey; *p* < 0.001) and PrPKO mice (Tukey; *p* < 0.001). The extent of sCHI in all experimental groups was also assessed by immunostaining for the neuronal markers MAP2 (Fig. 12) and myelin basic protein (MBP) (Fig. 13). While there was a localized decrease in the amount of each of these markers immediately inside the injury zone, there was no evidence of widespread decreases of these markers throughout the cortex in any experimental group (Fig. 14f, g).

## Discussion

TBI causes cellular injury to neuronal and nonneuronal cells. This results in the activation of many pathways and the triggering of numerous neuropathological and pathophysiological processes. Trauma results in a damaged blood-brain barrier, ionic imbalances, energy depletion, and cell death. Neurotrauma initiates an increase in extracellular glutamate and intra-axonal calcium levels. Increased calcium activates calpains, caspases, and phosphatases that trigger the cleavage of neurofilaments and α-spectrin, which leads to the disruption of the cytoskeleton and cell death. TBI could play a major role in the etiology of AD and CTE years after the neurotrauma event [47]. The TBI-initiated neuropathological alterations linked to AD and CTE include, but are not limited to, cerebral accumulation of misfolded protein aggregates, synaptic dysfunction, and neuronal loss, along with behavioral impairments. Thus, TBI appears to trigger and exacerbate some of the pathological processes associated with tauopathies (i.e., AD, CTE), in particular, the formation and accumulation of misfolded protein aggregates composed of amyloid-beta (Aβ) and Tau. Taken together, the previous reports on AD and the findings reported in this manuscript on sCHI suggests that although many pathophysiological processes are activated as a result of TBI, the PrP^C^-Tau pathology link may play an influential role in the long-term consequences.

PrP^C^ is expressed most abundantly in the brain, but has also been found in non-neuronal tissues [33, 62]. Although PrPKO mice have been reported to have only minor alterations in immune function, PrP^C^ is up-regulated during T cell activation and suggests an important, but unclear, role in T-cell function [18]. Studies have also suggested that PrP^C^ is required for continued stem cell generation of the haematopoietic system. Since PrP^C^ is predominantly expressed in the CNS, the major site of prion disease pathology, identifying and characterizing the function of PrP^C^ in neurons has been a major area of research. Although PrPKO mice do not have any gross neuropathological changes, even when neuronal PrP^C^ is knocked out postnatally, they do have subtle abnormalities in synaptic transmission, hippocampal morphology, circadian rhythms, cognition and seizures. Additional neuron-associated roles for PrP^C^ include a metal binding protein such as copper, both anti-apoptotic and pro-apoptotic protein, cell signaling, neuronal morphology and cell adhesion. PrP^C^ may also function in oxidative stress homeostasis [61] and play a role in maintaining long-term memory [62].

The primary function of Tau is to facilitate assembly and maintenance of microtubules in neuronal axons, allowing transport of cellular macromolecules [52]. Phosphorylation plays a major role in regulating the normal physiologic function of Tau and Tau neuropathogenesis. Tau phosphorylation is a complex process which can occur by many different intracellular pathways involving kinases (serine/threonine kinases, tyrosine kinases), phosphatases and other post-translational modifications. There are 85 putative phosphorylation sites on the longest Tau isoform, and more than 20 Ser/Thr kinases, as well as tyrosine kinases, have been shown to phosphorylate Tau in vitro and in vivo. The list of kinases and phosphatases that actively phosphorylate and de-phosphorylate Tau is frequently being updated [16, 17, 27, 45, 58]. Numerous Tau-associated phosphorylation sites and pathways associated with Tau dysfunction and neurodegeneration have been reported for AD [16, 17, 44].

There are numerous pathways and mechanisms associated with Tau phosphorylation and resulting in neurodegeneration. Neurofibrillary tangles (NFTs), a hallmark of AD, are composed of paired helical filaments consisting of abnormally hyperphosphorylated Tau. Cavallini et al. [6] studied the regulation and phosphorylation of Tau in human neuroblastoma cells and primary cortical neurons. They identified GSK3α, GSK3β, and MAPK13 as the most active Tau kinases phosphorylating Tau at 4 of the pathological-associated sites (epitopes pSer202, pThr231, pSer235, and pSer396/404).

Fyn is a 59 kDa protein belonging to the Src family of tyrosine kinases. The biological functions of Fyn are diverse and include T-cell receptor signaling, cell division and adhesion, synaptic function and plasticity and CNS myelination [19, 25, 30, 50, 55]. This phosphorylation is critical for LTP and long-term depression that are linked to learning and memory [26, 28]. Activation of Fyn’s synaptic function activity can have the undesired effect of rendering neurons vulnerable to synaptotoxicity. A reduction of Fyn activation has the opposite effect which may be neuroprotective although excessive inhibition may lead to impaired LTP and poor cognition.

Previous findings [23] demonstrated that extracellular Aβo binds PrP^C^ with high affinity, activating an intracellular signaling cascade coupled to the protein tyrosine kinase Fyn. Thus, the ability of Aβo to activate Fyn is dependent on the presence of PrP^C^ and requires mGluR5 [53]. This suggests that in AD, extracellular Aβo trigger neuronal signal transduction from PrP^C^ to mGluR5 to Fyn kinase. Fyn activation, in turn, hyperphosphorylates and mislocalizes Tau protein in the dendritic spines, leading to destabilized microtubules, which produce NFTs and the cognitive impairment characteristic of AD patients. Overexpressing Fyn was found to accelerate synapse loss and the onset of cognitive impairment in a transgenic AD mouse models [8]. In addition, the Aβo-PrP^C^ interaction has been linked to memory impairments in multiple AD mouse models [20]. The blocking of binding between Aβo and PrP^C^ is currently being tested as a therapeutic approach to prevent or treat AD pathology [9, 20, 60].

Tau may play a critical role in mediating downstream neurodegeneration in AD [37]. Similar to studies of Aβo, studies of Tau have implicated Fyn mechanistically in AD. Fyn physically associates with Tau, and can phosphorylate tyrosine residues, including Tyr18, near the amino terminus [3, 4]. Tyr18 is also phosphorylated in NFTs in human AD brain, suggesting a possible clinical relevance [24]. Activation of Fyn by the Aβo-PrP^C^ complex also leads to downstream Tau phosphorylation [22].

CTE is generally distinguishable from other tauopathies and AD because it consists of a unique distribution of pathological changes throughout the brain [29]. Interestingly, autopsy brain samples from CTE-diagnosed athletes and military veterans, and others affiliated with some form of TBI, display accumulation of misfolded protein aggregates. Tau aggregates are the most abundant with additional amyloid-beta (Aβ) and TDP-43 aggregates in some cases [29]. Furthermore, Tau accumulation is the predominant feature of CTE-associated rmTBI, while Aβ deposits are not very conspicuous, unless in more severe forms of TBI. Currently, a conclusive CTE diagnosis can only be made at autopsy. NFTs, neuropil threads, and astrocytic tangles form in an irregular distribution and heavy density in the frontal and temporal cortices [42, 47, 48]. Despite these pathological differences in CTE and AD, the Tau isoforms that are hyperphosphorylated remain identical between CTE and AD.

As reported in this manuscript, our data suggests that PrP^C^ is important in mediating pathology following TBI. We have found that following sCHI, PrPKO mice did not display an increase in P-Tau expression when examined biochemically (brain and blood) and neuropathologically by IHC. These mice also did not exhibit cognitive deficits compared to their sham-treated controls. This is in contrast to WT and Tga20 mice in which increases in brain and blood P-Tau concentrations after sCHI were demonstrated and found to be dependent on the levels of PrP^C^ expression. Furthermore, WT and Tga20 mice showed cognitive deficits post sCHI which varied according to their increased P-Tau concentrations. In addition, neurodegeneration-associated astrocytosis and gliosis, as measured biochemically by the levels of GFAP in brain and blood, increased after sCHI in all three mouse strains whether or not PrP^C^ was expressed or changes in P-Tau concentrations were detected. All of these changes in protein levels, modifications and cognition were unaffected by the administration of the calpain inhibitor, SNJ-1945. Overall, our studies suggest that the generation of P-Tau following severe TBI is independent of calpain activity but requires PrP^C^ leading to cognitive deficits. Therefore the mechanism(s) associated with neurodegeneration and cognitive deficits resulting from severe TBI may, in part, involve a similar mechanism as associated with AD. Our studies of P-Tau focused on the pSer202 epitope. Following the screening of a limited number of various P-Tau epitopes, we found that the pSer202 epitope is relatively highly reactive in rodent P-Tau. However, future studies examining additional P-Tau sites would be worthwhile.

TBI can affect anyone and can enhance the risk of certain brain diseases. Head insults can alter the brain, producing pathology such as toxic aggregates, inflammation, and structural alterations. Thus, brain trauma can result in disease-causing and disease-accelerating capabilities, ultimately being a main reason for these affected individuals to develop a more severe neurodegenerative disorder.

Despite the complexity of TBI, AD, and CTE, an obvious feature indicating a common mechanism is the presence of misfolded proteins: Aβ and Tau. As observed largely from human and animal studies, Aβ and Tau accumulation originate following a TBI event and progress with age, thereby potentially playing a part in the etiology and pathogenesis of AD and CTE. Exploring the mechanisms of TBI and its link to brain disorders such as AD and CTE may provide a better understanding of the etiopathogenesis of neurodegenerative diseases.

## Conclusions

1. Following sCHI, increasing levels of biochemically assayed T-Tau and P-Tau in the brains were associated with the PrP^C^ expression levels.
2. Following sCHI, biochemically assayed P-Tau concentration in the blood was associated with PrP^C^ expression in the absence of significant T-Tau changes.
3. The concentrations of biochemically measured GFAP rose quickly after sCHI and then progressively decreased. This effect was independent of PrP^C^ expression.
4. Changes in sCHI-induced biomarker concentrations was independent of gender and calpain hyperactivation.
5. Biochemically assayed biomarker concentrations correlated with quantitative immunohistochemistry.
6. A relationship was demonstrated between the presence/absence of PrP^C^, the levels of P-Tau and cognitive dysfunction.

## Abbreviations

AD: Alzheimer’s disease
a-EIMAF: Rolling circle amplification linked to enhanced immunoassay using multi-arrayed fiberoptics
APA: Active place avoidance
Aβ: Amyloid-beta
Aβo: Amyloid-β oligomers
CNS: Central nervous system
CTE: Chronic traumatic encephalopathy
EIMAF: Enhanced immunoassay using multi-arrayed fiberoptics
GFAP: Glial fibrillary acidic protein
IHC: Immunohistochemistry
LTP: Long term potentiation
Mab: Monoclonal antibody
NFTs: Neurofibrillary tangles
PFA: Paraformaldehyde
PrP^C^: Cellular prion protein
PrPKO: PrP^C^ knockout mice
P-Tau: Phosphorylated Tau
rmTBI: Repetitive mild traumatic brain injury
sCHI: Severe closed head injury
TBI: Traumatic brain injury
TDP-43: 43 kDa TAR DNA binding protein
Tga20: PrP^C^ overexpressing mice
T-Tau: Total Tau
WT: Wild-type mice
